# Supplementary material for: Modeling the microbial contribution to human energy balance using the Digestion, Absorption, and Microbial Metabolism (DAMM) model
Source: PLoS One. 2026 May 27;21(5):e0347668. doi: 10.1371/journal.pone.0347668 (PMC13215556; doi:10.1371/journal.pone.0347668)
Supplement: S1 File — Includes supplemental tables and figures as well as the GitHub code repository link, and a section detailing an example of yield calculations for methanogens. (DOCX) [file pone.0347668.s001.docx]

Supplemental information on DAMM mathematical model

# Supplemental Information on DAMM model availability

The model is stored in this GitHub code repository: <https://github.com/tldavi25/DAMM-Model>.

# Supplemental Information Tables

**S1 Table:**  **Colonic transit time and methanogen data.** DAMM inputs for measured colonic transit time (CTT) [1] and calculated initial methanogen biomass concentration from *mcrA* qPCR measurements [2]. The starred value is a removed outlier where minimal methane was measured despite the presence of methanogens within the feces.

| **ID (Treatment)** | **X_M_^0^ [10^3^ gCOD]** | **CTT [d]** |
| --- | --- | --- |
| P01 (MBD) | 1.9 | 0.9 |
| P01 (WD) | 0.8 | 0.9 |
| P02 (MBD) | 0 | 3.0 |
| P02 (WD) | 0 | 1.6 |
| P03 (MBD) | 2.7 | 0.9 |
| P03 (WD) | 1.3 | 2.4 |
| P04 (MBD) | 0 | 1.5 |
| P04 (WD) | 0.1 | 1.0 |
| P05 (MBD) | 2.2 | 1.0 |
| P05 (WD) | 1.3 | 3.9 |
| P06 (MBD) | 0.1 | 2.7 |
| P06 (WD) | 0 | 3.8 |
| P07 (MBD) | 0 | 0.9 |
| P07 (WD) | 0 | 1.4 |
| P08 (MBD) | 0 | 0.7 |
| P08 (WD) | 0 | 1.2 |
| P09 (MBD) | 8.2 | 1.4 |
| P09 (WD) | 3.4 | 2.5 |
| P10 (MBD) | 0 | 1.0 |
| P10 (WD) | 0 | 1.2 |
| P11 (MBD) | 2.0 | 1.7 |
| P11 (WD) | 1.8 | 2.7 |
| P12 (MBD) | 3.6 | 1.9 |
| P12 (WD) | 0.6 | 0.7 |
| P13 (MBD) | 0.8^*^ | 0.5 |
| P13 (WD) | 1.7 | 0.5 |
| P14 (MBD) | 0 | 0.83 |
| P14 (WD) | 0 | 0.9 |
| P15 (MBD) | 0 | 0.6 |
| P15 (WD) | 0 | 0.7 |
| P16 (MBD) | 0 | 0.1 |
| P16 (WD) | 0 | 1.7 |
| P17 (MBD) | 0 | 1.1 |
| P17 (WD) | 0 | 0.6 |

**S2 Table:** **DAMM Digestivity Constants.** Digestivity constants by nutrient for absorption within the UGI.

| **Macronutrient** | **Nutrient** | **α** | **Source** |
| --- | --- | --- | --- |
| carbohydrates | Sucrose | 0.975 | [3] |
|  | Glucose | 0.975 |  |
|  | Fructose | 0.975 |  |
|  | Lactose | 0.975 |  |
|  | Maltose | 0.975 |  |
|  | Galactose | 0.975 |  |
|  | Starch | 0.925 |  |
|  | Cellulose | 0.08 | [4] |
| fats | Butyric acid | 1.000 | [5] |
|  | Caproic acid | 1.000 |  |
|  | Caprylic acid | 0.000 |  |
|  | Capric acid | 0.974 |  |
|  | Lauric acid | 0.873 |  |
|  | Myristic acid | 0.778 |  |
|  | Palmitic acid | 0.208 |  |
|  | Stearic acid | 0.000 |  |
|  | Arachidic acid | 0.000 |  |
|  | Behenic acid | 0.767 |  |
|  | Palmitoleic acid | 0.939 |  |
|  | Oleic acid | 0.000 |  |
|  | Linoleic acid | 0.812 |  |
|  | α-Linolenic acid | 0.985 |  |
|  | Arachidonic acid | 0.799 |  |
|  | EPA | 0.359 |  |
|  | DPA | 0.000 |  |
|  | DHA | 0.000 |  |
| proteins | Alanine | 0.881 | [4] |
|  | Arginine | 0.902 |  |
|  | Asparagine | 0.873 |  |
|  | Cystine | 0.855 |  |
|  | Glutamine | 0.936 |  |
|  | Glycine | 0.715 |  |
|  | Histidine | 0.902 |  |
|  | Isoleucine | 0.909 |  |
|  | Leucine | 0.919 |  |
|  | Lysine | 0.936 |  |
|  | Methionine | 0.931 |  |
|  | Phenylalanine | 0.896 |  |
|  | Proline | 0.899 |  |
|  | Serine | 0.865 |  |
|  | Threonine | 0.847 |  |
|  | Tryptophan | 0.767 |  |
|  | Tyrosine | 0.889 |  |
|  | Valine | 0.897 |  |

**S3 Table:** **DAMM Stoichiometry Table.** Stoichiometry matrix for the hydrolysis and fermentation of the carbohydrates and proteins within the LGI tract compartment. The matrix units are in gCOD unless specified as grams of nitrogen (gN) instead. Valerate is excluded v from this table, but it is present in the stoichiometry matrix.

| Electron donors: | Donor | Microbial biomass | Hydrogen sulfide | Ammonia (gN) | Acetate | Propionate | Butyrate | Methane | Hydrogen |
| --- | --- | --- | --- | --- | --- | --- | --- | --- | --- |
| Unspecified Carb | -1.00 | 0.20 | 0.00 | -0.02 | 0.42 | 0.23 | 0.16 | 0.00 | 0.00 |
| Sucrose | -1.00 | 0.20 | 0.00 | -0.02 | 0.42 | 0.23 | 0.16 | 0.00 | 0.00 |
| Glucose (dextrose) | -1.00 | 0.20 | 0.00 | -0.02 | 0.42 | 0.23 | 0.16 | 0.00 | 0.00 |
| Fructose | -1.00 | 0.20 | 0.00 | -0.02 | 0.42 | 0.23 | 0.16 | 0.00 | 0.00 |
| Lactose | -1.00 | 0.20 | 0.00 | -0.02 | 0.42 | 0.23 | 0.16 | 0.00 | 0.00 |
| Maltose | -1.00 | 0.20 | 0.00 | -0.02 | 0.42 | 0.23 | 0.16 | 0.00 | 0.00 |
| Galactose | -1.00 | 0.20 | 0.00 | -0.02 | 0.42 | 0.23 | 0.16 | 0.00 | 0.00 |
| Starch | -1.00 | 0.20 | 0.00 | -0.02 | 0.42 | 0.23 | 0.16 | 0.00 | 0.00 |
| Fiber, total dietary | -1.00 | 0.20 | 0.00 | -0.02 | 0.42 | 0.23 | 0.16 | 0.00 | 0.00 |
| Unspecified Protein | -1.00 | 0.20 | 0.00 | 0.11 | 0.35 | 0.09 | 0.35 | 0.00 | 0.00 |
| Alanine | -1.00 | 0.20 | 0.00 | 0.13 | 0.00 | 0.00 | 0.00 | 0.00 | 0.80 |
| Arginine | -1.00 | 0.22 | 0.00 | 0.30 | 0.15 | 0.25 | 0.00 | 0.00 | -0.09 |
| Aspartic acid | -1.00 | 0.20 | 0.00 | 0.20 | 0.53 | 0.00 | 0.00 | 0.00 | 0.27 |
| Cystine | -1.00 | 0.17 | 0.17 | 0.13 | 0.59 | 0.00 | 0.00 | 0.00 | 0.07 |
| Glutamic acid | -1.00 | 0.20 | 0.00 | 0.18 | 0.36 | 0.00 | 0.44 | 0.00 | 0.00 |
| Glycine | -1.00 | 0.27 | 0.00 | 0.27 | 1.07 | 0.00 | 0.00 | 0.00 | -0.33 |
| Histidine | -1.00 | 0.20 | 0.00 | 0.25 | 0.36 | 0.00 | 0.44 | 0.00 | 0.00 |
| Isoleucine | -1.00 | 0.20 | 0.00 | 0.04 | 0.00 | 0.00 | 0.00 | 0.00 | 0.11 |
| Leucine | -1.00 | 0.20 | 0.00 | 0.04 | 0.00 | 0.00 | 0.00 | 0.00 | 0.11 |
| Lysine | -1.00 | 0.20 | 0.00 | 0.11 | 0.23 | 0.00 | 0.57 | 0.00 | 0.00 |
| Methionine | -1.00 | 0.18 | 0.08 | 0.06 | 0.00 | 0.64 | 0.00 | 0.00 | 0.09 |
| Phenylalanine | -1.00 | 0.20 | 0.00 | 0.03 | 0.00 | 0.00 | 0.00 | 0.00 | 0.80 |
| Proline | -1.00 | 0.22 | 0.00 | 0.06 | 0.15 | 0.25 | 0.00 | 0.00 | -0.09 |
| Serine | -1.00 | 0.20 | 0.00 | 0.16 | 0.64 | 0.00 | 0.00 | 0.00 | 0.16 |
| Threonine | -1.00 | 0.22 | 0.00 | 0.09 | 0.40 | 0.00 | 0.50 | 0.00 | -0.13 |
| Tryptophan | -1.00 | 0.20 | 0.00 | 0.06 | 0.00 | 0.00 | 0.00 | 0.00 | 0.80 |
| Tyrosine | -1.00 | 0.20 | 0.00 | 0.03 | 0.00 | 0.70 | 0.00 | 0.00 | 0.10 |
| Valine | -1.00 | 0.20 | 0.00 | 0.06 | 0.00 | 0.00 | 0.67 | 0.00 | 0.13 |
| Hydrogen |  | 1.02 | 0.00 | -0.01 | -1.02 | 0.00 | 0.00 | 1.00 | -1.00 |
| Influent | Y_inf_/Θ_x_ | 0.00 | 0.00 | 0.00 | 0.00 | 0.00 | 0.00 | 0.00 | 0.00 |
| Effluent | -Y_eff_/Θ_x_ | -Y_eff_/Θ_x_ | -Y_eff_/Θ_x_ | -Y_eff_/Θ_x_ | -Y_eff_/Θ_x_ | -Y_eff_/Θ_x_ | -Y_eff_/Θ_x_ | -Y_eff_/Θ_x_ | -Y_eff_/Θ_x_ |

**S4 Table:** **Acronym table.** Acronyms/variables for both supplemental and main text.

| **Acronym/Variable** | **Name** | **Unit** | **Description** |
| --- | --- | --- | --- |
| amt_food_ | Mass of consumed food item per day | g_food_ d^‑1^ | The amount of a food item consumed in a day |
| COD | Chemical oxygen demand | gCOD | An indicative measure of the amount of oxygen that can be consumed, directly proportional to the electron equivalents in a food’s carbon. |
| COD_biomass_ | Specific chemical oxygen demand for biomass | gCOD g_biomass_^‑1^ | COD per gram for a biomass for C_5­_H_7_O_2_N |
| COD_CH4_ | Methane chemical oxygen demand | gCOD d^‑1^ | Daily COD that leaves the human body as methane gas. |
| COD_f_ | Fecal chemical oxygen demand | gCOD d^‑1^ | Total daily amount of ingested COD that is expelled in the feces (can be specified as only from specific nutrient N) |
| COD_g_ | Gross chemical oxygen demand | gCOD d^‑1^ | Total daily amount of ingested COD from food (can be specific to a nutrient i.e. COD_g_^starch^ would be the total ingested COD of starch) |
| COD_m_ | Metabolizable chemical oxygen demand | gCOD d^‑1^ | Total daily amount of ingested COD that is metabolizable by the host (can be specified as only from UGI or LGI or a specific nutrient N) |
| COD_nutrient_ | Specific chemical oxygen demand of nutrient | gCOD g_nutrient_^-1^ | COD per gram for a specific nutrient calculated using chemical formula |
| CSTR | Continuously stirred reactor |  | A tank reactor characterized by a continuous flow of reactants into and products from the reaction system |
| CTT | Colonic transit time | d | Time duration it takes for ingested food to travel from the ileocecal valve to the anus |
| DAMM | Digestion, Absorption, and Microbial Metabolism |  | Mathematical model that tracks the progression of ingested macronutrients as they advance through the host’s digestive tract until they are subsequently absorbed and metabolized by organs and tissues or excreted – includes microbial contributions explicitly |
| E_g_ | Gross energy | kcal d^‑1^ | Total combustible energy within food items of a diet, usually expressed as a rate |
| E_m_ | Metabolizable energy | kcal d^‑1^ | Amount of energy available for human metabolic processes after absorption in the digestive tract, usually expressed as a rate |
| e_scfa abs_ | Efficiency of SCFA absorption | gCOD_abs_ gCOD^‑1^ | Proportion of SCFAs that are absorbed within the colon calculated using saturation model dependent on CTT |
| f_dry_ | Fraction of dry mass | g_dry wt_ g_wet wt_^‑1^ | Mass fraction of dry mass to wet mass |
| f_nutrient_ | Mass fraction of nutrient in food item | g_nutrient_ g_food_^‑1^ | The amount of a specific nutrient in a food item (e.g. the fraction of starch in potatoes) |
| f_org_ | Fraction of organic dry mass | g_org dry wt_ g_dry wt_^‑1^ | Mass fraction of organic dry mass to dry mass |
| k_C ferm_ | Carbohydrate fermentation coefficient | gCOD^-1^ d^-1^ | Second-order kinetic coefficient for fermentation of simple sugars like glucose or fructose |
| k_C hyd_ | Carbohydrate hydrolysis coefficient | gCOD^-1^ d^-1^ | Second-order kinetic coefficient for hydrolysis of complex carbohydrates like fiber or resistant starch |
| k_F abs_ | Fat absorption coefficient | d^‑1^ | First-order absorption coefficient for fat in the LGI |
| k_M_ | Methanogenesis coefficient | gCOD^-1^ d^-1^ | Second-order kinetic coefficient for methanogenesis |
| k_P hyd_ | Protein hydrolysis coefficient | gCOD^-1^ d^-1^ | Second-order kinetic coefficient for hydrolysis of proteins into amino acids |
| K_scfa abs_ | Half-saturation constant for SCFA abosrption | d | Half-saturation constant for absorption of SCFAs in the LGI |
| LGI | Lower gastrointestinal tract |  | Subsection of the gastrointestinal tract encompassing the passageway from the start of the colon to the anus |
| MBD | Microbial enhancer diet |  | One of two diets within the clinical trial with the same ratios of carbohydrates, proteins, and fats and the necessary calories to remain in energy balance, this diet was designed to increase the available substrates for the gut microbes |
| *mcrA* | Methyl coenzyme M reductase | copy # | Enzyme that catalyzes the final step in the formation of methane, also known as coenzyme-B sulfoethylthiotransferase |
| N | Nutrient | gCOD | A specific nutrient considered within the diet (e.g. starch, glucose, alanine, DHA, etc.) |
| N_copy_ | Gene copy number per cell | copy # cell^‑1^ | Typical number of copies for a specific gene (*mcrA* in this work) within a cell |
| qPCR | Quantitative polymerase chain reaction |  | Laboratory technology used for measuring DNA using polymerase chain reaction. |
| R^2^ | Coefficient of determination |  | Measure of how well observed outcomes are replicated by the model |
| R_ut_ | Utilization rate per day | gCOD d^‑1^ | Total utilization rate per day – included five processes: 1) protein hydrolysis and fermentation 2) carbohydrate hydrolysis and fermentation 3) methanogenesis 4) absorption of fat in colon and 5) absorption of SCFAs in colon |
| SCFA | Short-chain fatty acids |  | Fatty acids of two to four carbon atoms. In this work, we consider acetic, propionic, and butyric acid |
| SRT | Solids’ retention time | d | Time the solid fraction of the flow spends in a tank e.g. the amount of time the microbial biomass spends in the colon |
| UGI | Upper gastrointestinal tract |  | Subsection of the gastrointestinal tract encompassing the passageway from the mouth to the start of the colon, i.e. the esophagus, the stomach, and the small intestine |
| USDA | United States Department of Agriculture |  | Acronym for government department that created the food central database |
| WD | Western diet |  | One of two diets within the clinical trial with the same ratios of carbohydrates, proteins, and fats and and the necessary calories to remain in energy balance, this diet was designed to be deficient in microbiome-fermentable substrates |
| wt_cell_ | Wet weight per cell | g_wet wt_ cell^‑1^ | Average wet weight per cell – calculated using cell dimensions and density |
| X | Biomass | gCOD | Biomass within the colon |
| X_M_ Factor | Methanogen solids-concentration factor | cells_colon_ cells_feces_ | A factor calculated using the DAMM model that estimates the amount of methanogens in the colon relative to the amount measured in the feces |
| X_M_^0^ | Initial methanogen biomass in colon | gCOD | A starting amount of methanogen microbial biomass within the colon estimated using qPCR measurements in fecal samples |
| α_N_ | Absorption fraction of nutrient *N* | g_abs_ g_ingested_^-1^ | Fraction of absorbed mass within the UGI of the total ingested amount for specific nutrient |
| σ_est_ | Standard error of the estimate | gCOD d^‑1^ | Estimation of the accuracy of metabolizable COD predictions |
| τ | Kendall’s coefficient of concordance |  | Indicator of proportional bias between methods |

# Supplemental Information Figures


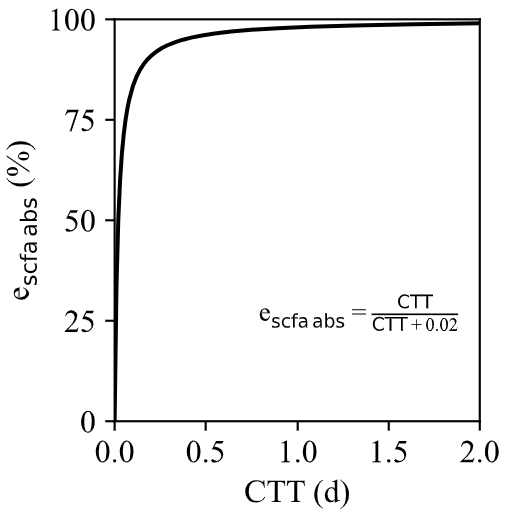


**S1 Fig:** **SCFA Absorption Saturation Model**

The saturation model (Eq. 12) for the absorption of SCFAs within the colon calculates the percentage of absorbed SCFAs for CTT up to 2 days.


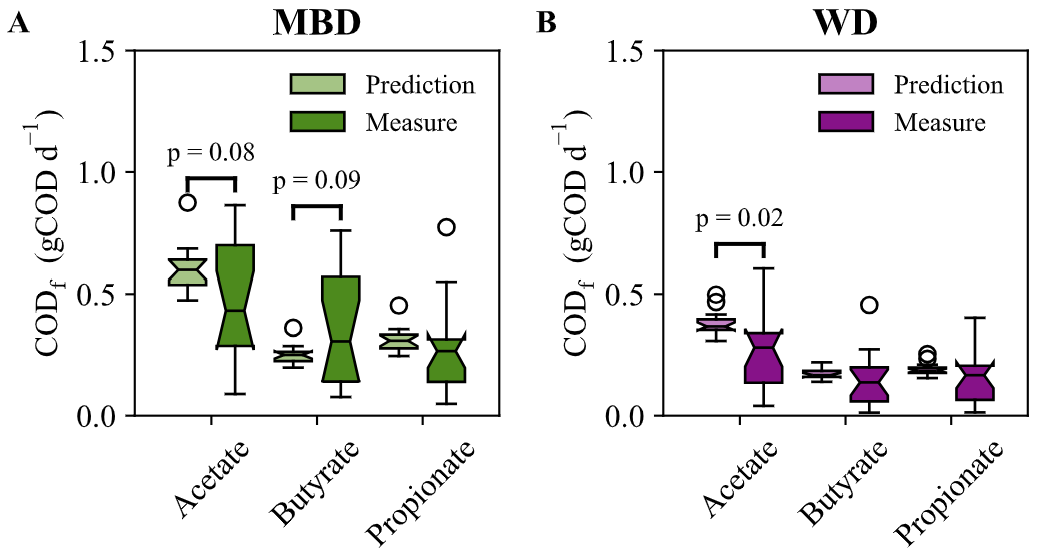


**S2 Fig: Predicted Fecal SCFAs with fixed 95% Absorption instead of Saturation Model**

A) Predictions for acetate, butyrate, and propionate fecal COD directly compared to the measurements for the microbial enhancer diet (MBD). B) Predictions for acetate, butyrate, and propionate fecal COD directly compared to the measurements for the western diet (WD). Statistical significance was determined using a t-test. All p-values > 0.3, except when labeled on the plot

# Supplemental Information Methods

## Methanogen Yield Calculations

We used the same half-reaction approach from Chapter 5 of Rittmann and McCarty [6] to develop the stoichiometry for all the other reactions as we did for methanogenesis; however, since we assumed the methanogens used acetate (CH_3_COOH) as a carbon source for the cell synthesis, while hydrogen (H_2_) was the electron donor for the energy production [7], the calculations were slightly more complicated and are summarized here.

The method balances half reactions for the electron donor for energy production (H_2_), the carbon source for cell synthesis (CH_3_COOH), the formation of microbial cells (methanogens – C_5_H_7_O_2_N), and the electron acceptor (CO_2_ reduced to CH_4_) by systematically weighing the Gibb’s free energy associated with the half reactions in Tables 5.3 to 5.5 in Rittmann and McCarty [6].

| **R_c_^synthesis^:** | $\frac{1}{4}CO_{2}+e^{-}+H^{+}\to\frac{1}{4}H_{2}O+\frac{1}{8}CH_{3}COOH$ | $\Delta{G_{cs}^{0}}^{'}=27.4\frac{kJ}{e^{-}}$ | (3) |
| --- | --- | --- | --- |
| **R_d_^energy^:** | $e^{-}+H^{+}\to\frac{1}{2}H_{2}$ | $\Delta{G_{d}^{0}}^{'}=39.9\frac{kJ}{e^{-}}$ | (4) |
| **R_cells_:** | $\frac{1}{20}NH_{4}^{+}+\frac{1}{20}HCO_{3}^{-}+\frac{1}{5}CO_{2}+e^{-}+H^{+}\to\frac{9}{20}H_{2}O+\frac{1}{20}C_{5}H_{7}O_{2}N$ | * | (5) |
| **R_a_:** | $\frac{1}{8}CO_{2}+e^{-}+H^{+}\to\frac{1}{4}H_{2}O+\frac{1}{8}CH_{4}$ | $\Delta{G_{a}^{0}}^{'}=-23.5\frac{kJ}{e^{-}}$ | (6) |

First, we calculated the energy required to convert the carbon source to activated acetate (∆G^0^’ = 30.9 kJ/e^-^), the common organic intermediate that cells use in synthesis.

| $\Delta G_{p}=30.9 \left[ \frac{kJ}{e^{-}} \right]- \Delta G_{cs}^{0^{'}}=30.9-27.4=3.5\frac{kJ}{e^{-}}$ | (1) |
| --- | --- |

In cells, activated acetate is converted to cellular carbon, which has an estimated energy of $\Delta G_{pc}=18.8\frac{kJ}{e^{-}}$ [6]. We assumed the energy-transfer efficiency was $\varepsilon=0.6$ We set $n=1$ because additional energy is required to convert acetate to activated acetate. Cell synthesis energy requirement was then calculated using Supplemental Information Equation 2.

| $\Delta G_{s}=\frac{\Delta G_{p}}{\varepsilon^{n}}+\frac{\Delta G_{pc}}{\varepsilon}=\frac{3.5}{0{.6}^{1}}+\frac{18.8}{0.6}=37.2\frac{kJ}{e^{-}}$ | (2) |
| --- | --- |

Then, using the listed Gibb’s free energy for the electron acceptor reaction, methane, and the electron donor for the energy production, hydrogen, we calculated the Gibb’s energy of the reaction.

| $\Delta G_{r}=\Delta{G_{a}^{0}}^{'}-\Delta{G_{d}^{0}}^{'}=-23.5-39.9=-63.4\frac{kJ}{e^{-}}$ | (7) |
| --- | --- |

Next, we use the calculated energy of reaction, energy of synthesis, and the assumed energy-transfer efficiency to get the amount of energy equivalents of the electron donor that must be oxidized (A):

| $A=-\frac{\frac{\Delta G_{p}}{\varepsilon^{n}}+\frac{\Delta G_{pc}}{\varepsilon}}{\varepsilon\Delta G_{r}}=-\frac{37.2}{0.6\left( -63.4 \right)}=0.98$ | (7) |
| --- | --- |

This amount of the electron donor consumed for energy (A equivalents) was then used to estimate the maximum fraction of electron equivalents used for synthesis (f_s_^0^) and the minimum fraction of electron equivalents for energy production (f_e_^0^).

| $f_{s}^{0}=\frac{1}{1+A}=\frac{1}{1+0.98}=0.51$ | (8) |
| --- | --- |
| $f_{e}^{0}=1-f_{s}^{0}=1-0.54=0.49$ | (9) |

Lastly, we created a balanced stoichiometry equation using the fractions for synthesis and energy production and the half reactions listed in Supplemental Information Equations 3-6 and then calculated the yield.

| $R=f_{e}{(R}_{a}-R_{d}^{energy})+f_{s}(R_{cells}-R_{c}^{synthesis})$  $H_{2}+0.26CH_{3}COOH+0.12NH_{4}^{+}+0.10HCO^{-}+0.15CO_{2}\to0.91H_{2}O+0.25CH_{4}+0.10C_{5}H_{7}O_{2}N$ | (10) |
| --- | --- |
| $Y=0.10 mol cells\left( 113\frac{g cells}{mol cells} \right)/ 1 mol H_{2}= 11.57\frac{g cells}{mol H_{2}}$ | (11) |

For a more detailed explanation on how to calculate stoichiometry equations and yields using this standardized procedure, please refer to the Rittmann and McCarty Environmental biotechnology textbook [6].

It may be useful to note that the stoichiometry table in Supplemental Table 2 is in gCOD not mols, so the stoichiometry coefficients in Supplemental Equation 10 are multiplied by the specific chemical oxygen demand, or the *COD* (gCOD mol^-1^) for each component.

# Supplemental Information References

[1] K. D. Corbin *et al.*, “Host-diet-gut microbiome interactions influence human energy balance: a randomized clinical trial,” *Nature Communications 2023 14:1*, vol. 14, no. 1, pp. 1–17, May 2023, doi: 10.1038/s41467-023-38778-x.

[2] B. Dirks *et al.*, “Methanogenesis associated with altered microbial production of short-chain fatty acids and human-host metabolizable energy,” *ISME J*, vol. 19, no. 1, p. 103, Jan. 2025, doi: 10.1093/ISMEJO/WRAF103.

[3] A. M. Stephen, A. C. Haddad, and S. F. Phillips, “Passage of carbohydrate into the colon. Direct measurements in humans,” *Gastroenterology*, vol. 85, no. 3, pp. 589–595, 1983, doi: 10.1016/0016-5085(83)90012-4.

[4] A. M. Rowan, P. J. Moughan, M. N. Wilson, K. Maher, and C. Tasman-Jones, “Comparison of the ileal and faecal digestibility of dietary amino acids in adult humans and evaluation of the pig as a model animal for digestion studies in man,” *British Journal of Nutrition*, vol. 71, no. 1, pp. 29–42, 1994, doi: 10.1079/bjn19940108.

[5] S. P. Ndou, E. Kiarie, M. C. Walsh, N. Ames, C. F. M de Lange, and C. M. Nyachoti, “Interactive effects of dietary fibre and lipid types modulate gastrointestinal flows and apparent digestibility of fatty acids in growing pigs,” *British Journal of Nutrition*, vol. 121, pp. 469–480, 2018, doi: 10.1017/S0007114518003434.

[6] B. E. Rittmann and P. L. McCarty, *Environmental biotechnology. Technology Guide: Principles and Applications*, 2nd ed. McGraw-Hill Book Co, 2020.

[7] B. S. Samuel *et al.*, “Genomic and metabolic adaptations of Methanobrevibacter smithii to the human gut,” *Proc Natl Acad Sci U S A*, vol. 104, no. 25, pp. 10643–10648, Jun. 2007, doi: 10.1073/PNAS.0704189104.
